# Understanding the Burden: Exploring Key Factors in Dementia Caregiving in Residential Care Homes

**DOI:** 10.1097/jnr.0000000000000690

**Published:** 2025-06-26

**Authors:** Yi-Heng CHEN, Li-Chan LIN, Pei-Hao CHEN, I-Hsuan LU

**Affiliations:** 1 Department of Nursing and Institute of Geriatric Welfare Technology and Science, Mackay Medical University, Taiwan; 2 Department of Nursing, Asia University, Taiwan; 3 Department of Neurology, MacKay Memorial Hospital, Taiwan; 4 Faculty of Public Health and Policy, London School of Hygiene and Tropical Medicine, United Kingdom

**Keywords:** caregiver burden, dementia, formal caregivers, aggression, residential care homes

## Abstract

**Background:**

The factors of influence on caregiver burden with regard to dementia care provided in residential care homes are multifaceted. Few studies in the literature have simultaneously investigated the variables related to residential care recipients with dementia and their formal caregivers.

**Purpose:**

This study was designed to assess caregiving burden in formal caregivers of residential care recipients with dementia and to identify significant predictors of this burden associated with these care recipients and their caregivers.

**Methods:**

This prospective cross-sectional study enrolled 206 registered nurses and nursing assistants working as formal caregivers, as well as 256 residents with dementia across 6 residential care homes. Structured questionnaires were used to collect data on the sociodemographic characteristics, attitudes, dementia care knowledge, and caregiver burden of nursing staff, as well as data on the sociodemographic characteristics, daily functional ability, cognitive functioning, and neuropsychiatric symptoms of residential care recipients with dementia.

**Results:**

The formal caregivers in this study reported experiencing mild to moderate care burden. Stepwise linear regression analysis identified prior dementia care training, confidence in care provision, and attitude in the formal caregivers and number of medications and agitation/aggression levels in the residents as significant predictors of caregiving burden, explaining 32% of the variance. Unexpectedly, the basic dementia care knowledge of the caregivers and the physical dependence and other neuropsychiatric symptoms of the residents were not identified as significant predictors of caregiver burden.

**Conclusions/Implications for Practice:**

The findings of this study underscore the critical importance of caregiver attitude, confidence, and coping skills in determining caregiving burden, noting that not all behavioral and psychological symptoms impact this burden equally. These insights emphasize the need to enhance confidence and positive attitudes in formal caregivers and to effectively manage residents’ aggressive behaviors through both pharmacological and nonpharmacological approaches to ameliorate the high caregiver burden associated with dementia care.

## Introduction

Population aging has led to a rapid increase in the number of older adults living with dementia and other functional limitations worldwide (World Health Organization [WHO], 2021). Dementia, characterized by an irreversible decline in cognitive and functional ability, is frequently accompanied by behavioral and psychological symptoms. As the progression of dementia and care needs escalate, institutionalization is an unavoidable outcome for many affected individuals (Aasmul et al., 2016). Nursing staff who work as formal caregivers in residential dementia care settings may face more challenging care issues and a higher workload than their counterparts in other health care settings (Roe et al., 2020).

Caring for residents with dementia during disease progression frequently imposes significant physical and psychological burdens on formal caregivers (Ornstein & Gaugler, 2012). These burdens may negatively impact these caregivers’ overall well-being and health and increase staff turnover rates, compromising the quality of care provided to residents (Ornstein & Gaugler, 2012; Sun et al., 2018). Identifying the factors that significantly contribute to these burdens is necessary to facilitate the design of targeted burden-alleviating interventions.

Despite the variations in research design and disease severity in prior research on this topic, their findings suggest several factors that may influence stress, distress, burden, and burnout in formal caregivers providing care to patients with dementia in long-term residential care facilities. These factors may be broadly grouped into 2 categories, namely caregiver-related factors and care-recipient–related factors. Although the age, sex, educational level, and working hours may not relate significantly to caregiving burden, longer experience in dementia care, more training in dementia care, and a more positive attitude towards dementia may be significant predictors of lower caregiving burden (Costello et al., 2019; Lee et al., 2013). In terms of residents, dementia severity, daily functional abilities, and behavioral disturbances have been identified as significant predictors of caregiver burden as well as potential contributors to caregiver mental health problems and burnout (Aasmul et al., 2016; Costello et al., 2019; Sun et al., 2018).

Previous studies in this field have elucidated the variables linked to the burden shouldered by formal caregivers. However, their conclusions are affected by limitations in the scope and methodology used in these studies. First, prior studies have mainly focused on either formal caregivers or residents with dementia and have not considered the potential influences arising from their mutual interaction, potentially overlooking important factors of influence on caregiving burden (Costello et al., 2019). Second, formal caregiver burden severity and its influencing factors in diverse residential dementia-care settings have been inadequately explored in the literature (Song & Oh, 2015; Sun et al., 2018). Third, in prior research, level of distress associated with each individual symptom has been used as the dependent variable rather than overall caregiver burden (Song & Oh, 2015; Van Duinen-Van Den Ijssel et al., 2018), which may fail to capture overall caregiver burden and the complex interplay between symptoms.

Developing strategies to improve the well-being and care quality provided by formal caregivers requires more comprehensive research on this topic. Therefore, this study was developed to assess the burden experienced by formal caregivers providing care to residents with dementia in residential care homes and to identify both caregiver and resident-related predictive variables of this burden. In addition, potential differences in caregiver burden predictors were examined for 2 different types of residential care home formats, that is, specialized dementia-care units and mixed care homes.

## Methods

A cross-sectional, prospective study using convenience sampling was conducted in 6 residential long-term care homes located in central and northern Taiwan from September 2019 to August 2020. Due to COVID-19 pandemic constraints, residential care homes and participants were selected based on their availability and willingness to participate during the specified time frame. These care home venues included both specialized dementia-care units and mixed-care home facilities. This research received ethical approval from a hospital-based ethical committee (18MMHIS174), and all participants provided signed informed consent before data collection.

### Participants

In this study, formal caregivers, including registered nurses (RNs) and nursing assistants (NAs), and residents with dementia were recruited from 3 specialized dementia-care units and 3 mixed-care homes. Specialized dementia-care units are specifically designed to accommodate dementia patients only, while mixed homes regularly provide care for older adults affected by dementia and/or frailty.

The inclusion criteria for formal caregivers were: (1) a minimum of one-months’ work experience in their current facility, (2) possession of a registered nurse license in Taiwan (RNs) or completion of the minimum mandatory training course (NAs), and (3) proficiency in either Mandarin or Taiwanese. The inclusion criteria for residents were having a diagnosis of dementia from a neurologist or psychiatrist and being currently under the care of a formal caregiver.

The minimum sample size for formal caregivers in this study was determined using G*Power analysis specifically for multiple linear regression. Using a medium effect size (*R* ^*2*^= .336) for 13 initially identified predictors, a 0.95 confidence level (*α* = .05), and 0.80 power (β = 0.20) resulted in a minimum sample of 66 participants. Of the 212 eligible RNs and NAs, 206 agreed to participate. Six RNs were excluded because they did not complete the questionnaire during the data collection period due to heavy workload. Of the 286 eligible residents with dementia within the targeted facilities, 256 completed the structured questionnaire assessment with the assistance of formal caregivers and research assistants (RAs). The dropout rate for the residents was 10.49*%*, attributable to either discharge or hospitalization.

### Measurements

Information on caregiver characteristics, including demographics, work experience, average weekly working hours, professional training, and confidence in providing dementia care, was collected using a data sheet. The Approaches to Dementia Questionnaire (ADQ) and the Dementia Knowledge Assessment Tool Version 2 (DKAT2) were used to measure caregiver attitudes and knowledge toward dementia care. The Professional Care Team Burden Scale (PCTB) was used to evaluate the dependent variable (caregiver burden). For the residents with dementia enrolled as participants, sociodemographic, medical history, and current health status data were collected using a data sheet, while the Barthel Index, Mini-Mental State Examination (MMSE), and Neuropsychiatric Inventory (NPI) were employed to assess their physical functions, cognitive abilities, and behavioral and psychological symptoms.

### Measurement Instruments for Formal Caregivers

The ADQ, developed by Lintern (2001), is a 19-item questionnaire with 2 subscales (hope and person-centeredness) designed to assess caregiver attitudes toward individuals with dementia. Each item is scored on a 5-point Likert-type scale (1 = *strongly disagree* to 5 = *strongly agree*), with total possible scale scores ranging from 19 to 95 and higher scores indicating more positive attitudes. The internal reliability of the ADQ and 2 subscales was assessed as .78, .70 (hope), and .77 (person-centeredness), respectively (Keogh et al., 2020). Construct validity, established using factor analysis, explained 40.4*%* of variance (Lintern, 2001). The Chinese version has demonstrated good reliability (Cronbach’s alpha = .74) and a positive correlation with the Sense of Competence in Dementia Care Staff scale (*r* = .22; Zhao et al., 2022).

This DKAT2, developed by Toye et al. (2014) as a revision of the original Dementia Knowledge Assessment Tool, is designed to assess the basic knowledge of caregivers regarding dementia disease and care. The first version, DKAT1, includes 33 items intended for care workers, while the revised DKAT2 includes 21 items intended for both families and care workers. The DKAT2 differs from DKAT1 primarily in terms of improved clarity, faster completion time, and enhanced reliability. The DKAT2 comprises “yes,” “no,” and “not sure” response options, with a higher number of correct responses indicating greater knowledge. The internal consistency reliability of this scale was .79, and known group validity was acceptable (Resciniti et al., 2020). In this study, the Chinese version showed good reliability (Cronbach’s alpha = .77) and a significant positive correlation with the ADQ (*r* = .50). The PCTB, developed by Auer et al. (2015), is designed to assess the burden experienced by caregivers providing long-term care. It consists of 10 items scored on a 5-point Likert-type Scale (0 = *strongly disagree* to 4 = *strongly agree*), with total possible scores ranging from 0 to 40 and higher scores reflecting greater burden. The reliability of the total scale was .79, and construct validity was confirmed by factor analysis (Auer et al., 2015). In this study, the Chinese version showed a Cronbach’s alpha of .74, and its significant positive correlation with average weekly working hours confirmed its criterion validity.

### Measurement Instruments for Older Adult Residents With Dementia

The Barthel index, developed by Mahoney and Barthel (1965), is designed to assess the respondent’s level of independence and mobility in performing activities of daily living (ADL). This index consists of 10 items, with scores ranging from 0 to 100 and higher scores indicating less physical dependence. The Barthel Index has shown good reliability and validity, with satisfactory interrater reliability (Intraclass Correlation Coefficient [ICC] = .98), and correlations with other physical functioning measurements (*ρ* = .57–.88; dos Reis et al., 2022). The Chinese version has been validated in Taiwan, showing high interrater reliability (ICC = .94; Yang et al., 2022) and significant correlations with depression symptoms and subjective well-being (*ρ* = −.36 and .23, respectively; Zhang et al., 2022), supporting its criterion-related validity.

The MMSE, developed by Folstein et al. (1975), is designed to assess cognitive abilities. This instrument consists of 30 items, with a maximum total score of 30 and higher values indicating higher cognitive functioning. The commonly recommended score ranges are 18–23 for mild, 11–17 for moderate, and < 11 for severe cognitive problems (Sjölund et al., 2021). The MMSE was assessed with a high test-retest reliability (*r* = .83–.99) and strong concurrent validity using the verbal score of the Wechsler Adult Intelligence Scale (*r* = .78; Folstein et al., 1975). The Chinese version has previously been validated, showing good internal consistency and a positive correlation with the Montreal Cognitive Assessment (MoCA; *ρ* = .834; Jia et al., 2021).

The NPI, designed to assess the frequency and severity of disturbance behaviors and caregiver distress, consists of 12 items across 4 subscales: psychosis, agitation, mood and behavior (Cummings, 2020). The symptom intensity score for each neuropsychiatric symptom in the NPI was derived by computing the product of its frequency, rated on a scale ranging from 1 (*occasional*) to 4 (*very frequent*), and its severity, rated on a scale ranging from 1 (*mild*) to 3 (*severe*). Total possible scale scores range from 0 to 144, with higher values indicating more severe disturbances. High test-retest reliability (ICC = .99; Saari et al., 2022) and validity, evidenced by correlation with the MMSE (Kaufer et al., 2000), have previously been shown. The Chinese version has also demonstrated satisfactory interrater reliability (ICC >.90) and good correlation with the Behavioral Pathology in Alzheimer’s Disease Rating Scale (BEHAVE-AD; *γ* = .48–.77; Leung et al., 2001).

### Study Procedure

The study was initiated after approval was obtained from the Institutional Review Board, which verified the research protocol and ensured adherence to ethical standards. The Principal Investigator (PI) personally visited the 6 residential care homes to explain the study’s objectives and obtain consent for study implementation from the facility supervisor or their designated representative. Subsequently, the PI and RAs provided detailed information about the research to the formal caregivers and the families of residents with dementia and obtained their consent before proceeding with participant recruitment. During the formal caregivers’ designated rest time, the PI and RAs explained the study process and the instruments used to them, and then distributed the questionnaire survey. Upon completion, the formal caregivers returned the questionnaires and received gift cards as compensation for their participation. Trained RAs implemented the MMSE and ADL with the older adult residents with dementia. Then, after explaining how to precisely complete the NPI questionnaire, the residents’ primary caregivers (i.e., those designated nurses and nursing assistants who had been responsible for their care for the past 2 weeks) jointly evaluated the residents’ NPI responses to minimize potential subjective information bias.

### Statistical Analysis

All data analysis was conducted using SPSS Statistics for Mac, Version 26.0 (IBM Corp., Armonk, NY, USA). *p* <.05 was established as statistical significance. Descriptive statistics were employed to summarize the basic characteristics of the residents and formal caregivers, as well as of the key study variables. Internal consistency of the scales used was assessed using Cronbach’s alpha. Pearson’s correlation coefficient was calculated to examine the associations between formal caregiver burden and potential independent factors, which were derived from both resident-related and caregiver-related variables. The burden of each caregiver (Y variables) was matched with the corresponding resident-related independent variables (X variables), which were derived by averaging the demographic data, clinical conditions, MMSE, ADL, and NPI scores of the residents for whom they had primary care responsibility for over the past 2-week period. To investigate the predictors of caregiver burden, variables that demonstrated a statistically significant association with caregiver burden in univariate analysis were included in the subsequent stepwise linear regression analysis.

## Results

### Sample Characteristics

The characteristics of the formal caregivers and residents are presented in Table 1. The formal caregiver participants in this study included 55 nurses and 151 nursing assistants. Most (89.8%) were females, were of a mean age of 45.77 years, and had an average 6.55 years of work experience. Their average working time was 45.65 hours per week; nearly 90% had received dementia-care-related training, and 82*%* self-reported as being confident in providing care for older adults with dementia.

**Table 1:** Participant Characteristics

Characteristic	*n* (%)
**Formal caregivers (*N*=206)**	
Type	
Nurse	55 (26.7)
Nursing assistant	151 (73.3)
Age (years; mean and *SD*)	45.77 (12.45)
Sex	
Female	185 (89.8)
Male	21 (10.2)
Educational level	
No formal schooling	2 (1.0)
Primary school	11 (5.3)
Secondary school	31 (15.0)
Advanced level	60 (29.1)
Vocational nursing school	46 (22.3)
University	48 (23.3)
Graduate school	8 (3.9)
Working experience (years; mean and *SD*)	6.55 (2.24)
Average weekly working hours (mean and *SD*)	45.65 (9.29)
Prior training in dementia care	181 (87.9)
Confidence in providing dementia care	169 (82.0)
**Older adult residents with dementia (*N*=256)**	
Age (years; mean and *SD*)	80.91 (9.12)
Sex	
Female	161 (62.9)
Male	95 (37.1)
Marital status	
Married	95 (37.1)
Single	10 (3.9)
Deceased partner	128 (50.0)
Separated	2 (0.8)
Divorced	21 (8.2)
Educational level	
No formal schooling	50 (19.5)
Primary school	73 (28.5)
Secondary school	42 (16.4)
Advanced level	42 (16.4)
University	44 (17.2)
Graduate school	5 (2.0)
Frequency of visits	
No visitors	7 (2.7)
Over 6 months	17 (6.6)
Every 4~6 months	9 (3.5)
Every 1~3 months	40 (15.6)
1~2 times per month	82 (32.0)
1~2 times per week	88 (34.4)
3 times a week or more	13 (5.1)
Length of stay in current institution (years; mean and *SD*)	3.56 (3.43)

Two hundred fifty-six older adult residents with dementia receiving care from the participating formal caregivers were included in the final analysis exploring factors associated with formal caregiver burden. These residents were of a mean age of 80.91 years, had a mean length of stay in their institution of 3.56 (*SD* = 3.43) years. In addition, 62.9*%* were female, 19.5*%* had no formal education, and over 70% received visits from family or friends a minimum of 1~2 times per month.

### Mean Scores for Key Variables

Mean scores for key variables related to formal caregivers and residents (stratified by type of care home) are presented in Table 2. Overall, the respective mean scores for PCTB, ADQ, and DKAT2 were 13.01 (*SD* = 5.11), 65.10 (*SD* = 5.46), and 16.27 (*SD* = 2.81). The PCTB score was below the midpoint of the scale, while the ADQ and DKAT2 scores were above the midpoint, suggesting that, while formal caregivers experienced a mild to moderate burden of care, they exhibited a more than moderate level of knowledge and attitude toward dementia care. In terms of type of care home, the mean scores indicated that formal caregivers in specialized dementia-care units had a slightly higher care burden (13.39 vs 12.41) and significantly better dementia care knowledge (16.61 vs 15.72) than their peers working in mixed care homes.

**Table 2:** Mean Scores for Key Variables Related to Formal Caregivers and Residents

Key Variable	Mean (*SD*)			Mean (*SD*)
	Specialized Dementia-Care Units (*n* = 127)	Mixed Care Home (*n* = 79)	*t*	Overall (*N* = 206)
Formal caregivers				
PCTB	13.39 (5.81)	12.41 (3.68)	−1.35	13.01 (5.11)
ADQ	65.14 (5.74)	65.03 (5.01)	−0.15	65.10 (5.46)
DKAT2	16.61 (2.54)	15.72 (3.15)	−2.23*	16.27 (2.81)
Older adult residents with dementia				
No. medications	7.01 (1.61)	7.55 (1.13)	2.61*	7.22 (1.47)
No. medical tubes	0.02 (0.07)	0.06 (0.14)	2.57*	0.04 (0.10)
MMSE	8.40 (2.09)	9.97 (1.94)	5.35**	9.06 (7.00)
ADL	50.59 (8.28)	38.26 (6.23)	−11.38**	54.59 (31.24)
NPI_f x s_Total	22.00 (15.13)	23.24 (9.02)	0.66	22.47 (13.11)
NPI_f x s_Delusion	2.85 (1.61)	2.64 (1.39)	−0.94	2.77 (1.53)
NPI_f x s_Hallucination	1.61 (2.02)	1.64 (1.12)	0.13	1.62 (1.73)
NPI_f x s_Agitation/aggression	1.63 (1.38)	1.38 (1.13)	−1.40	1.53 (1.29)
NPI_f x s_Depression/dysphoria	1.87 (1.81)	2.25 (1.67)	1.50	2.02 (1.77)
NPI_f x s_Anxiety	2.47 (1.95)	2.88 (2.31)	1.38	2.63 (2.10)
NPI_f x s_Elation/euphoria	0.81 (0.92)	1.14 (1.34)	2.08*	0.94 (1.11)
NPI_f x s_Apathy/indifference	1.03 (0.99)	1.30 (1.15)	1.80	1.13 (1.06)
NPI_f x s_Disinhibition	1.58 (1.62)	2.43 (1.26)	3.98**	1.90 (1.55)
NPI_f x s_Irritability/lability	2.68 (1.72)	2.33 (1.13)	−1.61	2.54 (1.53)
NPI_f x s_Aberrant moter behavior	2.03 (1.16)	2.76 (1.50)	3.96**	2.31 (1.35)
NPI_f x s_Sleep and nightime behavior disorders	1.84 (1.43)	1.50 (2.03)	−1.42	1.71 (1.69)
NPI_f x s_Appetite and eating disorders	1.61 (1.18)	0.99 (1.10)	−3.77**	1.37 (1.19)

*Note.* ADL = activities of daily living; ADQ = Approaches to Dementia Questionnaire; DKAT2 = Dementia Knowledge Assessment Tool version 2; MMSE = Mini-Mental State Examination; NPI = Neuropsychiatric Inventory; PCTB = Professional Care Team Burden Scale.

*p < .05. **p < .01.

Regarding the resident participants, the mean numbers of medication and medical tubes were 7.22 (*SD* = 1.47) and 0.04 (*SD* = 0.10), respectively. The mean scores for MMSE and ADL of 9.06 (*SD* = 7.00) and 54.59 (*SD* = 31.24), respectively, indicate severe cognitive impairment and functional disability. Furthermore, 91.4% of these residents with dementia exhibited more than one behavioral or psychological symptom, with delusion, irritability, anxiety, and aberrant motor behaviors identified as the top-4 most common symptoms. The mean scores for the residents indicated that those in specialized dementia-care units, while having significantly poorer cognitive function (8.40 vs 9.97) and receiving significantly fewer medications (7.01 vs 7.55) and medical tubes (0.02 vs 0.06), exhibited significantly better physical function (50.59 vs 38.26) than their peers living in mixed care homes.

#### Correlations between formal caregiver burden and key variables

Correlations between formal caregiver burden and the major independent variables were examined, with variables demonstrating noteworthy correlations presented in Table 3. Weekly working hours, previous training experience in dementia care, confidence in providing dementia care, ADQ score, and DKAT2 score were all found to correlate significantly with formal caregiver care burden. The resident-related variables MMSE score and ADL score both correlated significantly and negatively with PCTB. Also, significant positive correlations were observed between formal caregiver burden and, respectively, number of medications, number of medical tubes, and 3 NPI items (delusion, agitation/aggression, sleep and nighttime behavior disorders). The direction of these correlations (positive / negative) was consistent with the expected outcomes.

**Table 3:** Correlations between Formal Caregiver Burden and Key Variables (*N*=206)

Variable	1	2	3	4	5	6	7	8	9	10	11	12	13
Formal caregivers													
1. PCTB	–	–	–	–	–	–	–	–	–	–	–	–	–
2. Average weekly working hours	.23**	–	–	–	–	–	–	–	–	–	–	–	–
3. Prior training in dementia care	−.33**	−.08	–	–	–	–	–	–	–	–	–	–	–
4. Confidence in providing dementia care	−.17*	.03	.25**	–	–	–	–	–	–	–	–	–	–
5. Approaches to Dementia Questionnaire	−.38**	−.28**	.11	.00	–	–	–	–	–	–	–	–	–
6. DKAT2	−.23**	−.28**	.12	−.06	.45**	–	–	–	–	–	–	–	–
Older adult residents with dementia													
7. No. medications	.31**	.10	−.12	.02	−.10	.06	–	–	–	–	–	–	–
8. Number of medical tubes	.33**	.16*	−.15*	.02	−.19**	−.03	.37**	–	–	–	–	–	–
9. MMSE	−.21**	−.11	−.12	−.21**	.12	−.07	−.29**	−.40**	–	–	–	–	–
10. Activities of daily living	−.25**	−.22**	.22**	.03	.20**	.21**	−.36**	−.60**	.14	–	–	–	–
11. NPI_f x s_Delusion	.18**	.27**	−.02	−.01	−.27**	−.15*	−.15*	.32**	−.28**	−.30**	–	–	–
12. NPI_f x s_Agitation/aggression	.16*	.12	.07	.07	−.13	−.11	−.16*	.38**	−.50**	−.25**	.52**	–	–
13. NPI_f x s_Sleep and nighttime behavior disorders	.17*	.27**	−.06	−.05	−.06	.01	.12	.19**	.02	−.21**	.23**	.27**	–

*Note.* PCTB = Professional Care Team Burden Scale; DKAT2 = Dementia Knowledge Assessment Tool version 2; MMSE = Mini-Mental State Examination; NPI = Neuropsychiatric Inventory.

* *p* < .05. **p < .01.

#### Predictors of formal caregiver burden

All of the significant variables identified in the bivariate analysis were incorporated into the regression model as potential predictors of formal caregiver burden by type of dementia-care home (Table 4). Formal-caregiver-related variables, including previous training in dementia care, confidence in providing dementia care, attitudes toward dementia, and resident-related variables, including number of medications and NPI agitation/aggression, were each shown to significantly predict formal caregiver burden regardless of care home type. These independent variables accounted for 32% of the total variance in the overall model. In both models—for dementia specialized care units and mixed care homes—formal caregiver weekly working hours, confidence in providing dementia care, and attitudes toward dementia were consistently included in the final regression models. However, resident-related variables differed between the 2 models. In the specialized dementia-care home model, average number of medications and the presence of medical tubes significantly predicted PCTB, while in the mixed care home model, only MMSE was found to significantly impact formal caregiver care burden. The predictors in the specialized dementia-care unit and mixed care home models explained 51% and 28% of the variance, respectively. Notably, DKAT2 and ADL were not included in any of the 3 models and, unexpectedly, formal caregivers’ weekly working hours were shown to correlate negatively with care burden in the mixed-care home model.

**Table 4:** Multivariable Regression Analysis of Formal Caregiver Burden, by Type of Care Home

Independent Variable	Specialized Dementia-Care Units (*N* = 127)		Mixed Care Home (*N* = 79)		Overall (*N* = 206)	
	β	95% CI	β	95% CI	β	95% CI
Formal caregivers						
Average weekly working hours	0.19*	[0.03, 0.24]	−0.26*	[−1.53, −0.20]	–	–
Prior training in dementia care	–	–	−0.27*	[−4.65, −0.50]	−0.24*	[−5.65, −1.92]
Confidence in providing dementia care	−0.14*	[−4.31, −0.20]	−0.26*	[−4.10, −0.40]	−0.13*	[−3.27, −0.14]
ADQ	−0.26**	[−0.40, −0.13]	−0.22*	[−0.31, −0.01]	−0.30**	[−0.39, −0.17]
Older adult residents with dementia						
Number of medications	0.26**	[0.49, 1.41]	–	–	0.28**	[0.58, 1.39]
Number of medical tubes	0.33**	[14.69, 40.17]	–	–	–	–
MMSE	–	–	−0.21*	[−0.78, −0.02]	–	–
NPI_f x s_Agitation/aggression	–	–	–	–	0.19**	[0.30, 1.22]
Adjusted *R* ^*2*^	–	51%	–	28%	–	32%

*Note*. ADQ = Approaches to Dementia Questionnaire; CI = confidence interval; MMSE = Mini-Mental State Examination; NPI = Neuropsychiatric Inventory; β = standardized beta coefficient.

* *p* < .05. **p < .01.

## Discussion

The findings reveal that the formal caregiver participants generally experienced a mild to moderate level of burden, with those in specialized dementia-care units reporting a slightly higher burden but demonstrating significantly better dementia care knowledge than their peers in mixed care homes. Regardless of care home type, factors including previous training in dementia care, confidence in providing dementia care, and attitudes toward dementia among caregivers and number of medications used and NPI agitation/aggression among residents were identified as significant predictors of caregiver burden. However, surprisingly, basic dementia care knowledge in caregivers and physical dependence in residents, along with other types of behavioral and psychological symptoms, fell short of the significance necessary to be included in the overall model. Slight differences were observed between the specialized dementia-care home model and the mixed care home model in terms of variables associated with caregivers and residents with dementia.

Consistent with a previous study (Costello et al., 2019), this study demonstrated that caring for older adults with dementia imposes a mild to moderate burden on caregivers. Although seemingly manageable, this level of burden can still lead to psychological health issues for caregivers and adversely affect the quality of dementia care provided (Ornstein & Gaugler, 2012). Therefore, early identification and intervention to reduce or alleviate caregiver burden may be expected to help prevent adverse consequences for both residents and formal caregivers. Furthermore, although falling short of statistical significance, the formal caregivers in specialized dementia-care units reported a higher mean level of burden than their peers in mixed care homes. In this study, residents in specialized dementia-care units reported being better able to perform daily tasks but had poorer cognitive function than those living in mixed care homes, with respective ADL scores of 50.59 and 38.26 and respective MMSE scores of 8.4 and 9.97. This higher burden is likely attributable to caregivers in specialized dementia-care units being exclusively responsible for older adult residents with dementia, which implies they consistently face a cluster of stressful challenges (e.g., dealing with uncooperative residents, understaffing due to high turnover, communication difficulties leading to poor therapeutic interactions; Van Duinen-Van Den Ijssel et al., 2018). The demands of addressing these dementia-specific challenges likely exceed the burdens associated with providing general daily living assistance, likely resulting in a greater strain on caregivers working in specialized dementia-care units.

In examining caregiver-related predictors of caregiver burden, the findings of this study both agree with and diverge from the findings of prior research. Caring for residents with dementia is challenging, and adequate related training and adopting positive attitudes toward dementia care have been previously recognized as crucial factors in alleviating caregiver burden (Gerritsen et al., 2019; Lee et al., 2013). Gerritsen et al. found that caregivers who hold hopeful and person-centered attitudes are more effective in addressing and controlling residents’ challenging behaviors, enhancing the quality of caregiver-resident relationships and promoting psychological well-being in both residents and caregivers. However, contrary to expectations (Dietrich et al., 2014; Lee et al., 2013), caregiver level of knowledge about dementia care did not enter into any of the final 3 models. Moreover, having previous training experience in dementia care did not qualify for the specialized dementia-care units model. Also, an inverse correlation was observed between caregiver weekly working hours and caregiving burden in the mixed care home model. Three plausible explanations for these findings are: (1) While formal caregivers in both specialized dementia-care units and mixed care homes possess adequate dementia-care knowledge, tackling more challenging tasks in caring only for residents with dementia may rely more heavily on problem-solving experience, positive attitude, and confidence in care management, (2) Formal caregivers in specialized dementia-care units may accumulate substantial dementia-specific knowledge through regular and intensive interactions with residents, making previous training less impactful on their caregiving burden, and (3) A significant percentage (17.80%) of the formal caregivers in mixed care homes are part-time employees, who may lack adequate disease-specific preparation and thus contribute to higher caregiving burdens. Ongoing in-service education, attitude and empathy training, and situational exercises are recommended, particularly for part-time formal caregivers.

As for resident-based predictors of caregiver burden, the overall model identified 2, namely: number of medications used and agitation/aggressive behavior. Other factors, including cognitive function and number of medical tubes, varied in significance based on residential care home type. Several prior studies have established a strong correlation between aggressive behavior and caregiver burden, highlighting its distressing impact in terms of fostering an unsafe work environment and increasing physical and psychological risks for caregivers (Ornstein & Gaugler, 2012; Song & Oh, 2015; Van Duinen-Van Den Ijssel et al., 2018). In clinical settings, antipsychotics, anxiolytics, and antidepressants are frequently prescribed to manage dementia-related behavioral and psychological symptoms. In this study, up to 75% of residents with dementia received these medications. However, the potential for severe side effects and adverse reactions from these medications may exacerbate caregiver burden. Considering these findings, it is vital across different dementia care settings to enhance caregiver training in aggressive behavior management and optimize the administration of psychotropic medications. These efforts may significantly mitigate caregiver burden while improving the overall quality of dementia care.

The generally recognized primary patient-related factors contributing to caregiver burden in the context of dementia care are physical dependency, cognitive impairment, and behavioral and psychological symptoms. However, despite this, these factors were only partially supported as predictors in this study. Specifically, a significant correlation was not found between impaired daily functioning and caregiver burden. This result, although disagreeing with most existing research, aligns with Sun et al. (2018), who posited that professional caregivers in institutional settings feel less burdened by physically dependent individuals because of their specific expertise and familiarity with their residents’ characteristics and needs.

In assessing caregiver burden across care home types, cognitive impairment was a significant factor only in the mixed care home model. However, the specialized dementia-care unit model included not only number of medications but also number of medical tubes. This discrepancy may reflect that mixed care homes accommodate more residents who are severely disabled and/or tube-reliant, implying that caregivers in these settings may possess the requisite related skills to manage these cases, resulting in a lower perceived burden. Conversely, dementia-specific units have a higher tendency to transfer out high-dependency residents and thus typically have fewer residents requiring medical tubes such as urinary catheters, nasogastric tubes, and endotracheal tubes. Infrequent caregiver exposure to tube-related care in these units may thus result in higher perceived burdens when such care is required.

In terms of behavioral and psychological symptoms, only agitation/aggression was included as a significant predictor in the overall model. Although various types of dementia-related behavioral and psychological symptoms may cause distress to caregivers and increase care burden, aggressive behaviors pose a particular challenge because of the potential for self-harm, harm to others, and immediate danger. These factors may significantly affect caregiver safety, caregiver-resident interactions, and care quality, thus exacerbating perceived burden (Holst & Skär, 2017). This may explain why agitation/aggression emerged as a significant contributor in this model.

### Limitations

Several limitations should be considered when interpreting the results of this study. Firstly, multiple regression analysis may not fully capture the direct and indirect relationships among variables. Due to the limited amount of foundational research, the primary goal of this study was to identify key predictive variables and assess their direct impact on caregiver burden. In future studies on this topic, structural equation modeling should be employed to more clearly explain both direct and indirect effects. Secondly, relying on self-report questionnaires for both “caregiver burden” and “NPI” may introduce biases such as social desirability bias. Incorporating multiple data sources, for example, behavioral observations and caregiver interviews, may mitigate this limitation. Thirdly, although this study considered all of the significant predictors identified in the literature, the level of explained variance reached only 32%, suggesting other important factors have yet to be considered. These factors may include caregivers’ mental and physical health, level of social support received, and frequency of engaging residents in therapeutic activities, as well as dementia residents’ comorbidities and current communication abilities. These and other potentially relevant factors should be considered to more comprehensively understand caregiver burden. Lastly, the convenience sampling approach used (due to COVID-19 pandemic restrictions) may have introduced selection bias and limited external validity. Future research should employ cluster random sampling based on facility characteristics to enhance generalizability and provide a more comprehensive understanding of caregiver burden across different care settings.

### Conclusions

The results of this study underscore the need to mitigate the burden experienced by formal caregivers of older adults with dementia living in residential care homes. Although basic dementia knowledge has been identified in prior research as a predictive factor of caregiver burden, the findings of this study reveal caregiver attitudes, confidence, and coping skills informed by this knowledge to be critical factors determining caregiving burden. Moreover, this research showed that not all behavioral and psychological symptoms predict caregiver burden equally. Specifically, only aggressive behaviors linked to self-harm or harming others were identified as significant predictors. Targeted interventions, including situational training to enhance the attitudes, confidence, and coping skills of caregivers as well as strategies to address psychotropic medication usage and the management of aggressive behaviors in residents, will be necessary to effectively reduce caregiver burden and improve the quality of care provided to older adult residents with dementia.
